# Translation factor mRNA granules direct protein synthetic capacity to regions of polarized growth

**DOI:** 10.1083/jcb.201704019

**Published:** 2019-03-15

**Authors:** Mariavittoria Pizzinga, Christian Bates, Jennifer Lui, Gabriella Forte, Fabián Morales-Polanco, Emma Linney, Barbora Knotkova, Beverley Wilson, Clara A. Solari, Luke E. Berchowitz, Paula Portela, Mark P. Ashe

**Affiliations:** 1 Division of Molecular and Cellular Function, School of Biological Sciences, Faculty of Biology, Medicine and Health, The University of Manchester, Manchester Academic Health Science Centre, Manchester, UK; 2 Universidad de Buenos Aires, Instituto de Química Biológica de la Facultad de Ciencias Exactas y Naturales-Consejo Nacional de Investigaciones Científicas y Técnicas, Buenos Aires, Argentina; 3 Department of Genetics and Development, Hammer Health Sciences Center, Columbia University Medical Center, New York, NY

## Abstract

mRNA localization serves key functions in localized protein production, making it critical that the translation machinery itself is present at these locations. Here we show that translation factor mRNAs are localized to distinct granules within yeast cells. In contrast to many messenger RNP granules, such as processing bodies and stress granules, which contain translationally repressed mRNAs, these granules harbor translated mRNAs under active growth conditions. The granules require Pab1p for their integrity and are inherited by developing daughter cells in a She2p/She3p-dependent manner. These results point to a model where roughly half the mRNA for certain translation factors is specifically directed in granules or translation factories toward the tip of the developing daughter cell, where protein synthesis is most heavily required, which has particular implications for filamentous forms of growth. Such a feedforward mechanism would ensure adequate provision of the translation machinery where it is to be needed most over the coming growth cycle.

## Abstract (precis)

Pizzinga et al. show that mRNAs encoding a range of translation factors are localized to granules that get transported into the yeast daughter cell using the She2p/She3p machinery. This likely supports an intensification of protein synthetic activity to facilitate apical polarized growth.

## Introduction

mRNA localization can regulate spatiotemporal protein production to modulate a variety of critical physiological functions, including cell differentiation, polarization, and protein targeting to organelles/membranes (Holt and Bullock, 2009; Pizzinga and Ashe, 2014). Key localized mRNAs involved in establishing oocyte/embryonic polarity were among the first examples discovered. For instance, *β-actin* mRNA in ascidian embryos (Jeffery et al., 1983), *Vg1* in *Xenopus laevis* oocytes (Melton, 1987) and *bicoid* mRNA in *Drosophila melanogaster* oocytes (Berleth et al., 1988). Further specific localized mRNAs were found in the dendritic and/or axonal regions of neuronal cells, where they can provide the flexibility of structure and function required for synaptic plasticity (Garner et al., 1988; Miyashiro et al., 1994). Even in unicellular eukaryotes like yeast, mRNAs such as *ASH1* have been found to confer polarity between the mother and daughter cells (Long et al., 1997; Takizawa et al., 1997).

More recent assessments of mRNA localization suggest that, rather than being restricted to a handful of mRNAs, localization is remarkably widespread. In the *Drosophila* embryo, ∼70% of expressed mRNAs are localized in some manner (Lécuyer et al., 2007), while large numbers of localized mRNAs are present in the *Drosophila* ovary (Jambor et al., 2015), neuronal axon growth cones (Zivraj et al., 2010), and dendrites (Cajigas et al., 2012). Even in yeast, mRNA localization is much more commonplace than previously anticipated: mRNAs encoding peroxisomal, mitochondrial, and ER proteins, as well as mRNAs for general cytoplasmic proteins, are localized (Schmid et al., 2006; Zipor et al., 2009; Gadir et al., 2011; Fundakowski et al., 2012; Lui et al., 2014).

Studies at the mRNA-specific level have uncovered a number of key principles that resonate across many mRNA localization systems. The “prototype” mRNA in yeast studies was the *ASH1* mRNA, which localizes to the tip of the daughter cell to specifically repress mating type switching (Singer-Krüger and Jansen, 2014). *ASH1* mRNA localization relies upon actin cables and a specific myosin, Myo4p. In addition, the RNA-binding protein She2p interacts with the mRNA and, via the She3p scaffold, targets the mRNA to Myo4p (Singer-Krüger and Jansen, 2014). Cytoskeletal elements and motor proteins have been identified as common features of many mRNA localization mechanisms (López de Heredia and Jansen, 2004).

*ASH1* mRNA also highlights another key feature of many mRNA localization systems. That is, since inappropriate expression of Ash1p compromises the difference between the mother and daughter cells, the system is wholly reliant upon *ASH1* translational repression during mRNA transit. Similar tight translational regulation during transit has been identified for mRNAs in other systems such as morphogenetic gradient formation in *Drosophila* oocytes/embryos (St Johnston, 2005; Lasko, 2012). Equally, these systems rely upon specific translation derepression once an mRNA reaches its final destination (Besse and Ephrussi, 2008). In the case of *ASH1* mRNA, two mechanisms of translational derepression have been proposed involving Puf6p and Khd1p, respectively (Paquin et al., 2007; Deng et al., 2008).

Translation repression can also occur at a more global level—for instance, in response to stress (Spriggs et al., 2010; Simpson and Ashe, 2012). Such widespread repression has defined consequences in terms of mRNA localization: translationally repressed mRNA can transit to mRNA processing bodies (PBs) or stress granules (SGs; Kedersha et al., 2000; Brengues et al., 2005; Hoyle et al., 2007; Mollet et al., 2008; Simpson et al., 2014). PBs house many mRNA decay factors and have been considered as sites of mRNA turnover (Jain and Parker, 2013), although more recent studies in human cells favor a more dominant role for PBs in mRNA storage (Hubstenberger et al., 2017). SGs harbor a variety of RNA-binding proteins/translation factors and are thought of as sites of mRNA storage or triage (Anderson and Kedersha, 2008; Buchan and Parker, 2009). Recent studies have shown that these bodies adopt a more dynamic liquid structure than previously appreciated, such that enzymatic activities and protein refolding might be conceivable within the body (Aguzzi and Altmeyer, 2016; Mitrea and Kriwacki, 2016; Sfakianos et al., 2016). Both PBs and SGs can be induced by cellular stresses that bring about the robust repression of translation initiation (Kedersha et al., 2005; Teixeira et al., 2005; Wilczynska et al., 2005; Hoyle et al., 2007).

Recently, we have found that mRNAs can localize to granules even in rapidly growing cells and, at least for granules harboring glycolytic mRNAs, active mRNA translation occurs at these sites (Lui et al., 2014). Therefore, such mRNA granules might represent the kind of liquid, dynamic structure described above. Intriguingly, these glycolytic mRNA granules also appear to seed PB formation after stress and might represent sites where the fate of similar classes of mRNA is coordinated (Lui et al., 2014).

In this current paper, we have investigated the localization of another mRNA class in actively growing cells. We find that translation factor mRNAs localize to mRNA granules that are different to those carrying glycolytic mRNAs; they are fewer in number and display distinct inheritance patterns. Indeed, translation factor mRNA granules are specifically inherited by daughter cells and appear to play a role in focusing translational activity to sites of polarized growth. Overall, the protein synthetic capacity of a cell accumulates at specific sites via the localization of key mRNAs to facilitate polarized growth.

## Results

### Translation factor mRNAs are localized in actively growing yeast

Our previous work has established that two yeast glycolytic mRNAs, *PDC1* and *ENO2,* localize to and are translated in granules during active cell growth (Lui et al., 2014). Using *MS2*-tagging of endogenous mRNAs (the m-TAG system) and FISH, these mRNAs were shown to colocalize to 10–20 granules per cell. Following stress, the granules coalesced, then recruited PB components (Lui et al., 2014). In these studies, the *TIF1* mRNA, encoding the translation initiation factor eIF4A, was also identified as localized to granules, but at reduced frequency (fewer than five granules per cell; Lui et al., 2014).

**Figure 1.**
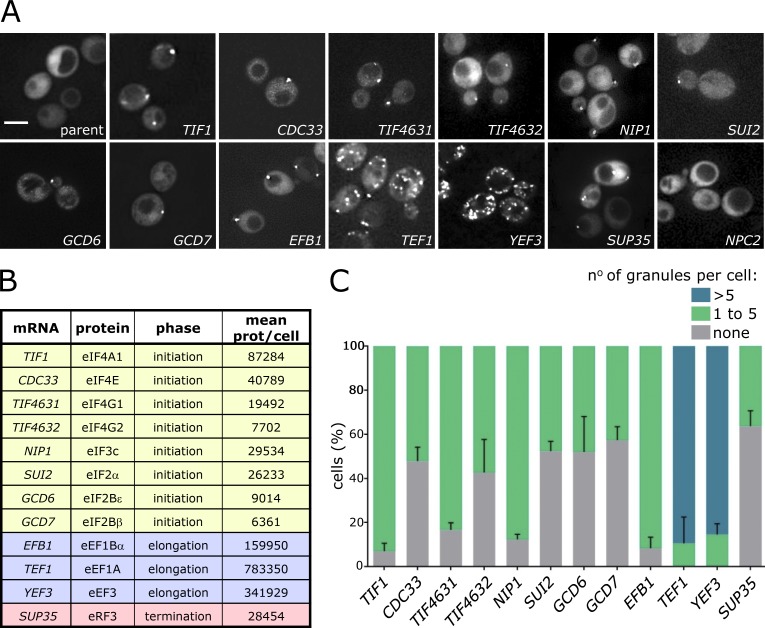
**Translation factor mRNAs localize to cytoplasmic granules in exponentially growing *S. cerevisiae*. (A)** Z-stacked images of strains expressing the labeled *MS2*-tagged mRNAs and the MS2 coat protein GFP fusion (MS2-CP-GFP). Bar, 4 µm. **(B)** The tagged mRNAs, proteins they encode, translation phase they function in, and mean protein (prot.) number per cell are listed (from Ho et al., 2018). **(C)** Chart showing the percentage of cells with one to five, more than five, or no granules per cell. Error bars = +SD (*n* = 3, >100 cells per repeat).

To study translation factor mRNA localization further, a range of mRNAs were selected and tagged using the m-TAG system. The selected mRNAs produce proteins with a range of abundances that participate in all three phases of translation: initiation, elongation, and termination (Fig. 1 B). The m-TAG technique involves the precise addition of *MS2* stem loops into the 3′ UTR of genes at their genomic loci, then coexpression of GFP fused to the MS2 coat protein (Haim-Vilmovsky and Gerst, 2009). Similar MS2-based GFP tethering systems have been widely used in yeast and other cells to study many aspects of RNA biology. A key advantage is that this technique allows mRNA localization to be studied in live cells (Buxbaum et al., 2015).

Intriguingly, all the investigated translation factor mRNAs localize to granules in unstressed cells (Fig. 1 A). Critically, under the active growth conditions used, PBs and SGs are not evident (Lui et al., 2014; see Fig. 3 D), suggesting that the mRNA localization does not relate to the stress-dependent formation of PBs or SGs (Teixeira et al., 2005; Hoyle et al., 2007; Grousl et al., 2009; Buchan et al., 2011; Iwaki and Izawa, 2012; Shah et al., 2016). Importantly, these translation factor mRNA localization patterns do not represent the norm, as many other mRNAs have a broad cytoplasmic localization (Lui et al., 2014): *NPC2* serves as a control here to illustrate this point (Fig. 1 A).

Even though all the translation factor mRNAs are localized, some variation in the pattern and number of mRNA granules per cell is evident (Fig. 1, A and C). Most of the mRNAs, including those encoding translation initiation factors, the eRF3 (*SUP35*) translation termination factor and the eEF1Bα (*EFB1*) elongation factor localize to fewer than five granules per cell (Fig. 1, A and C). In contrast, the two other tested elongation factor mRNAs, eEF1A (*TEF1*) and eEF3 (*YEF3*), localize to ∼10–20 granules per cell. This higher number of mRNA granules is more similar to that observed for the two yeast glycolytic mRNAs (Lui et al., 2014). When expression profiles were evaluated using the SPELL algorithm (version 2.0.3r71; Hibbs et al., 2007), which compares expression profiles across a plethora of transcriptomic experiments to identify similarly regulated genes, the translation elongation factor genes were identified as more similar to glycolytic genes than to genes encoding the rest of the translation machinery. It therefore seems that the expression of these translation elongation factor mRNAs is coregulated with mRNAs of the glycolytic pathway.

It is also noticeable that in terms of mRNA levels, the *TEF1* (eEF1A) and *YEF3* (eEF3) mRNAs are the most abundant tested (Fig. S1 A). This highlights the possibility that mRNA abundance plays a role in the propensity of an mRNA to enter granules. However, the abundance measurements for other mRNAs do not equate with their localization. For instance, mRNA abundance can vary from relatively low (*TIF4631* (eIF4G1) and *TIF4632* (eIF4G2) mRNAs) to *TIF1* (eIF4A1) mRNA, which is nearly as abundant as the translation elongation factor mRNAs (Fig. S1 A). Even though there is a large difference between these extremes, the pattern of mRNA localization is remarkably similar. This is suggestive that both the presence and pattern of an mRNA within RNA granules are not merely reflective of its overall abundance.

Recent studies have highlighted the potential impact of *MS2* stem loop addition to mRNAs in terms of mRNA fate (Garcia and Parker, 2015, 2016; Haimovich et al., 2016; Heinrich et al., 2017). In our own previous work, insertion of *MS2* stem loops decreased both *MFA2* and *PGK1* mRNA levels (Lui et al., 2014; Simpson et al., 2014). In this current study, we compared mRNAs from the m-TAG strains with *MS2* stem loops inserted and MS2-GFP fusion protein expressed to untagged mRNAs from the parent strain (Fig. S1 A). This analysis suggests that the MS2 system can have a complex and variable impact upon mRNA production and stability. For some mRNAs, such as *CDC33* (eIF4E), *EFB1* (eEF1Bα), *GCD6* (eIF2Bε), and *GCD7* (eIF2Bβ), the introduction of the MS2 system leads to a significant decrease in mRNA levels, whereas for others, such as *SUI2* (eIF2α), *TIF4631*, (eIF4G1), and *TIF4632* (eIF4G2), little significant effect is observed. In addition, there are a number of mRNAs where the MS2 system has an intermediate effect. It is unclear why the introduction of this system should reduce mRNA levels, and it is possible that multiple factors are at play. For instance, it is plausible that the introduction of the stem loops or just generally 3′ UTR alterations would affect mRNA production and 3′ end processing, or it could alter mRNA stability. This is not particularly surprising given that a well-established strategy for reducing yeast essential gene function is to insert a marker into a gene’s 3′ UTR (Breslow et al., 2008).

**Figure 2.**
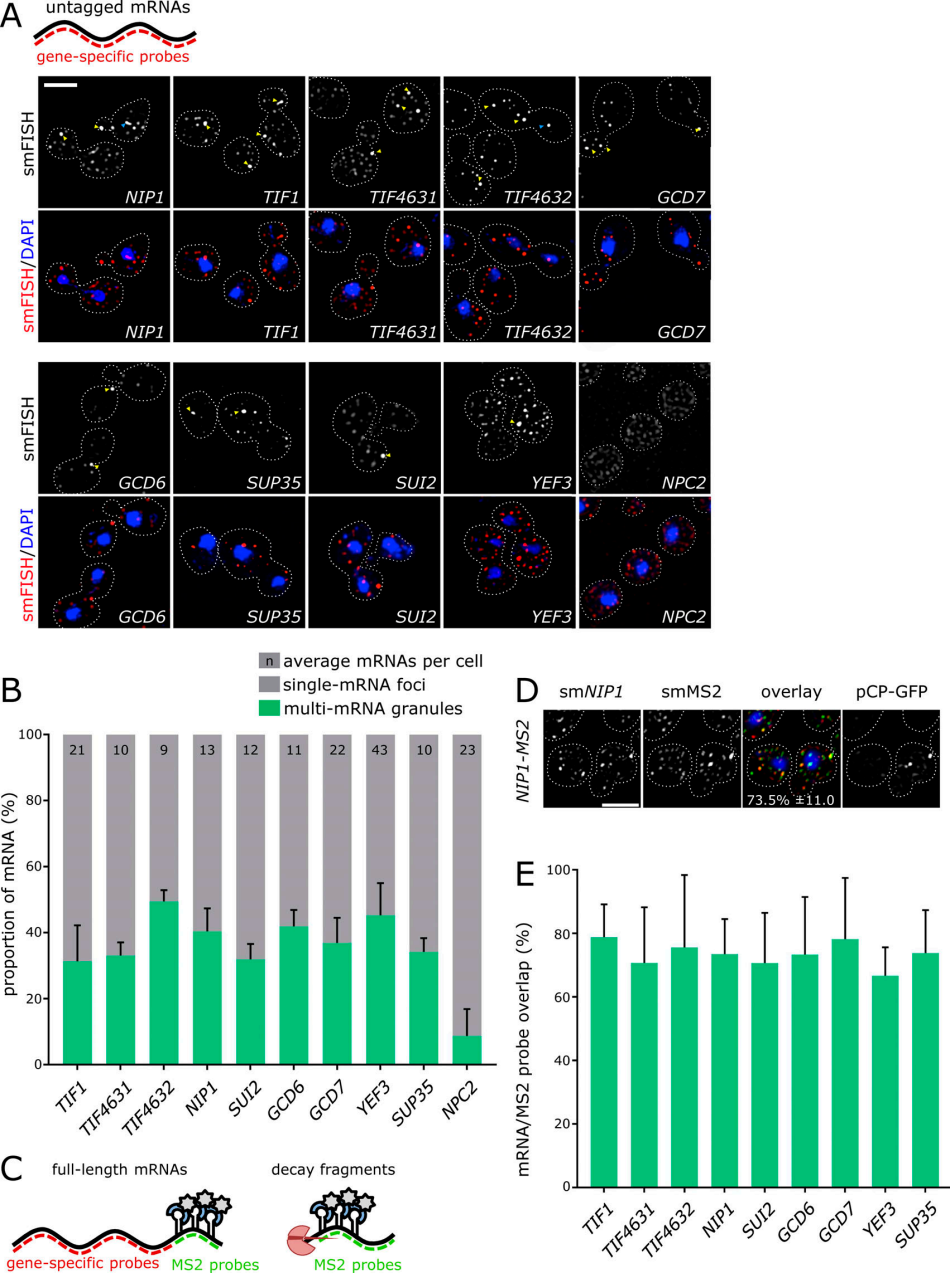
**smFISH recapitulates mRNA localization observed with the m-TAG system. (A)** Z-stacked smFISH images for the endogenous mRNAs indicated. **(B)** Bar chart showing mRNA proportion in either single mRNA foci (<2.5 mRNAs per spot), or multi-mRNA foci (>2.5 mRNAs per spot). **(C)** Diagram depicting the use of probes to either the gene body or *MS2* region, such that MS2-CP-GFP signal arising from full-length mRNAs (left), or 3′ decay fragment aggregates (right), is discernible. **(D)** Z-stacked image of a strain expressing *MS2*-tagged *NIP1*, visualizing the gene body (smNIP1), *MS2* loops (smMS2), and MS2-CP-GFP signal. **(E)** Bar chart depicting the overlap between MS2-CP-GFP foci and gene body foci for the indicated *MS2* mRNAs, as indicated (*n* = 3, >50 cells per repeat). Error bars = +SD. Bars, 3 µm.

Given the variability in the effects caused by the MS2 system and concerns regarding the integrity of mRNAs bound by the MS2-GFP fusion protein, it was important to assess mRNA localization using another independent technique. In previous studies, we have used FISH to show that the m-TAG system can reflect the genuine localization of endogenous mRNAs in yeast (Lui et al., 2014). Here, we adapted a single molecule FISH (smFISH) technique (Tsanov et al., 2016) for use in yeast to generate a high-resolution profile of the location of endogenous translation factor mRNAs (Fig. 2 A). smFISH appears more sensitive than m-TAG as both large multi-mRNA granules and smaller single mRNA foci are observed (Fig. 2 A). Using smFISH, the number of large multi-mRNA granules per cell correlates well with the numbers of granules per cell obtained using the MS2 system (Fig. 2 A). Even for *YEF3* (eEF3), where many mRNA granules were observed with the MS2 system, numerous large mRNA granules were observed with smFISH. From the smFISH data, it is also possible to estimate the number of mRNA molecules per cell. Such estimates compare favorably with the number of mRNAs per cell calculated from two RNA-seq studies (Fig. S1, B and C; Lawless et al., 2016; Lahtvee et al., 2017). This analysis also reveals the number of mRNA molecules present in the large granules as a proportion of the total (Fig. 2 B). As a result, we conclude that roughly half of the translation factor mRNAs in each cell are in large granules; the other half are present as single molecules. Interestingly for the *NPC2* mRNA, which is not observed in granules using the MS2 system, a much lower proportion of total mRNA was present in large granules using smFISH. These data show that endogenous translation factor mRNAs localize to large cytoplasmic granules and that the number of large granules is similar to that observed using the MS2 system.

To further explore the relationship between the m-TAG and smFISH data, smFISH was performed in the m-TAG strains comparing the localization profile observed using mRNA body probes versus probes to the *MS2* stem loops (Fig. 2, C and D). This comparison reveals a high degree of overlap, with >75% of the *MS2* stem loop signal overlapping with signal from the mRNA body (Fig. 2, D and E). Furthermore, significant overlap was observed to the GFP signal generated from the MS2-GFP fusion that is expressed in these yeast strains (Fig. 2 D). It is also clear from this analysis that only the most intense foci from the smFISH contain discernible GFP signal from the MS2-GFP fusion protein (Fig. S1 E). These data support the interpretation that the MS2 system does not detect single molecule mRNAs and only reveals larger multi-mRNA granules, as the MS2-GFP fusion is only detected for granules that have higher smFISH signal intensity (Fig. S1, D and E). However, the key point of this experiment is that where signal from the MS2-GFP was identified, signal from the mRNA body was also evident (>90%). Therefore, it appears that the MS2 system can faithfully reproduce endogenous mRNA localization patterns and can report the presence of full-length mRNAs but, in our MS2 experiments, not at single molecule resolution. In summary, the smFISH experiments further support an important role for mRNA localization in determining the fate of mRNAs encoding components of the translation machinery.

### mRNA granules harbor a complex mix of translation factor mRNAs

**Figure 3.**
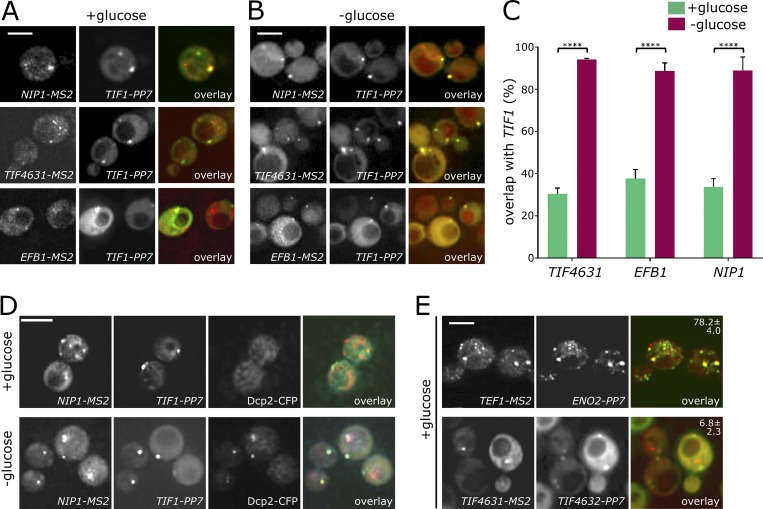
**Colocalization analysis of *MS2*- and *PP7*-tagged strains. (A and B)** Z-stacked images showing localization of *NIP1-MS2*, *TIF4631-MS2*, and *EFB1-MS2* (via MS2-CP-mCherry fusion) relative to *TIF1-PP7* (via PP7-CP-GFP fusion), actively growing (SCD media; A) and after 10-min glucose depletion (B). **(C)** Chart showing the percentage of *NIP1-MS2*, *EFB1-MS2*, or *TIF4631-MS2* granules colocalizing with *TIF1*-*PP7* granules in SCD media (green) and following glucose depletion (magenta). ****, P < 0.0001 (*n* = 3, >150 cells per repeat). Error bars = +SD. **(D)** Z-stacked images of *NIP1-MS2* and *TIF1*-*PP7* mRNAs relative to a PB marker Dcp2p-CFP in SCD media or after glucose depletion. **(E)** Z-stacked images of mRNAs: *TEF1-MS2* versus *ENO2-PP7* and *TIF4631-MS2* versus *TIF4632-PP7*. The percentage of *MS2*-tagged mRNA colocalizing with the PP7-tagged mRNA is indicated ± SD. Bars, 4 µm.

A key question is whether each multi-mRNA granule contains most translation factor mRNAs, or whether numerous granules exist with more variable mRNA composition. To address this question in live cells, we cross-compared the localization of different mRNAs using a *PP7* mRNA localization system in combination with the *MS2* system (Hocine et al., 2013; Lui et al., 2014). The PP7 system provides an analogous yet discrete mRNA localization system to MS2 in terms of specificity. Strains were generated with *PP7*-tagged *TIF1* (eIF4A1) mRNA, as well as another MS2-tagged translation factor mRNA. Two fusion proteins were coexpressed: PP7 coat protein–GFP and MS2 coat protein–mCherry. This allowed the simultaneous assessment of two different mRNAs within the same live cell (Fig. 3 A).

A comparison of the degree of overlap for the granules revealed that for each of the mRNAs *TIF4631* (eIF4G1), *NIP1* (eIF3c), and *EFB1* (eEF1Bα), ∼30% of mRNA granules also contained the *TIF1* (eIF4A1) mRNA (Fig. 3 C). Control experiments reveal that this colocalization is not due to crosstalk between the fluorescent channels (Fig. S2 A). We consider this overlap highly significant, as previously where we have assessed the overlap between a glycolytic mRNA (*PDC1*) and a translation factor mRNA (*TIF1*), we found no overlap (Lui et al., 2014). Moreover, comparison of the localization of *TIF4631* (eIF4G1) mRNA and *TIF4632* (eIF4G2) mRNA exhibited low levels of colocalization (Fig. 3 E). As well as highlighting the significance of the degree of overlap observed for other combinations (Fig. 3 A), this result indicates that not every mRNA is colocalized to the same set of granules.

In contrast to the mRNAs studied above, which do not colocalize with glycolytic mRNAs (Lui et al., 2014), there is significant colocalization between the elongation factor–encoding mRNAs (*TEF1*/*YEF3*) and the glycolytic mRNA *ENO2* (Fig. 3 E). Previously *ENO2* was shown to overlap almost perfectly with *PDC1* mRNA in granules that likely represent sites for coregulation of mRNAs with similar functions (Lui et al., 2014). The fact that translation elongation factor mRNAs also localize to the same granules further correlates with the transcriptional coregulation, mentioned above, that is evident from correlated expression profiles using the SPELL algorithm (version 2.0.3r71; Hibbs et al., 2007).

To corroborate the live cell colocalization studies, dual mRNA smFISH experiments were undertaken to investigate the colocalization of various endogenous mRNAs (Fig. S2). Once again, the degree of colocalization for *TIF1* versus *NIP1* and *TIF1* versus *TIF4631* was in the range 30–40% (Fig. S2), while much lower colocalization was observed for the *TIF4631* versus *TIF4632* mRNAs (Fig. S2). These smFISH results on endogenous mRNAs in fixed cells almost precisely parallel the observations made using the MS2/PP7 system in live cells.

Therefore, not every translation factor mRNA is contained in every granule; for instance, *TIF4631* and *TIF4632* mRNAs appear almost mutually exclusive. Instead, the results above support a model where a complex cocktail of translation factor mRNAs are housed within numerous mRNA granules.

### Translation factor mRNA granules coalesce to form PBs after stress

Previous work has suggested that mRNA granules carrying the *PDC1* and *ENO2* glycolytic mRNAs coalesce to seed the formation of PBs under glucose starvation conditions (Lui et al., 2014). To address the fate of the granules carrying translation factor mRNAs during PB formation, the PP7/MS2 colocalization strains were again used under rapid glucose depletion to induce PBs (Fig. 3 B). In this case, after 10 min glucose depletion, ∼90% of granules contained both *TIF1* mRNA and the relevant *MS2*-tagged mRNA (*TIF4631*, *NIP1*, or *EFB1*; Fig. 3 C). These data are consistent with a view that the translation factor mRNA granules also coalesce during the formation of PBs.

However, in order to directly assess whether these coalesced RNA granules are in fact PBs, the *NIP1* and *TIF1* mRNAs were evaluated with a CFP-tagged PB marker protein, Dcp2p (Fig. 3 D). Consistent with previous observations (Lui et al., 2014), the PB marker Dcp2p localizes broadly throughout the cytosol and does not overlap with the RNA granules in actively growing cells (Fig. 3 D). However, 10 min after glucose depletion, both the *TIF1* and *NIP1* mRNAs as well as Dcp2p are found in the same granules (Fig. 3 D). These experiments collectively show that the translation factor mRNA granules contribute to the formation of PBs in a similar manner to the RNA granules carrying glycolytic mRNAs (Lui et al., 2014).

### mRNA translation is a requirement for translation factor mRNA localization to granules

**Figure 4.**
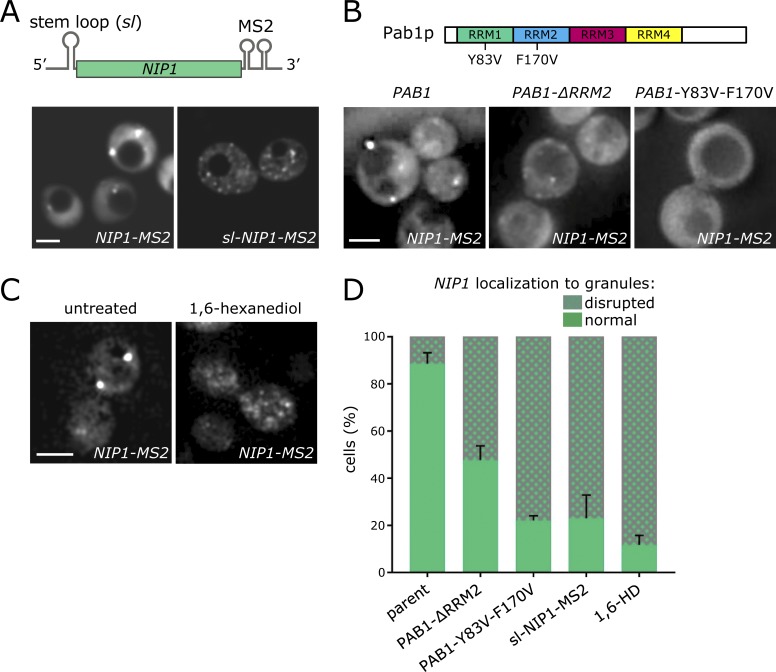
**Translation is required for correct localization of translation factor mRNAs to granules. (A)** Schematic of the *NIP1-MS2* construct with (*sl-NIP1-MS2*) or without (*NIP1-MS*2) a 5′ UTR stem loop to limit translation. Z-stacked images (below) of strains carrying these *NIP1* constructs. Bar, 5 µm. **(B)** Pab1p schematic detailing two point mutations, which impact upon translation initiation. Z-stacked images (below) for *NIP1-MS2* mRNA in *pab1Δ* strains bearing wild-type *PAB1*, *PAB1* lacking the RRM2 region, or *PAB1* with Y83V and F170V mutations. Bar, 4 µm. **(C)** Z-stacked images of *NIP1-MS2* mRNA in untreated or 1,6-hexanediol treated cells. Bar, 4 µm. **(D)** Bar chart depicting the impact of stem loop insertion, *PAB1* mutation, or hexanediol on *NIP1* mRNA granule integrity (*n* = 3, >100 cells per repeat). Error bars = +SD.

Previous work has suggested that mRNA granules can serve as mRNA translation sites in actively growing cells (Lui et al., 2014). To investigate whether translation of a specific mRNA affects its localization to granules, a well-characterized stem loop (ΔG value of −41 kcal/mol) was inserted into the *NIP1* mRNA 5′ UTR. This stem loop has previously been widely used to reduce translation of specific mRNAs by limiting scanning of the 43S preinitiation complex to the AUG START codon without impacting upon the stability of the mRNA (Vattem and Wek, 2004; Palam et al., 2011). In this case, the *MS2*-tagged *NIP1* mRNA was derived from a plasmid rather than the genome. A direct comparison of *NIP1* mRNA localization from a plasmid versus the genome revealed little difference in the localization to granules or number of granules per cell (Fig. S3). The insertion of a stem loop into the *NIP1* 5′ UTR significantly reduced the capacity of the *NIP1* mRNA to enter RNA granules (Fig. 4, A and D). Critically, the insertion of the stem loop did not significantly alter the expression level of the *NIP1* mRNA (0.165 ± 0.034 for *NIP1-MS2* versus 0.161 ± 0.022 for *sl-NIP1-MS2* relative to *ACT1* mRNA). These data suggest that translation of the *NIP1* mRNA might be important for its localization.

Further evidence that translation is important for mRNA localization comes from investigations of poly(A) binding protein, Pab1p. Pab1p is an RNA-binding protein with a characteristic set of four RNA recognition motifs (RRMs) and a C-terminal domain (Kessler and Sachs, 1998). Pab1p interacts with the mRNA poly(A) tail to elevate rates of translation initiation (Sachs et al., 1997). One mechanism by which Pab1p achieves this is via promotion of a “closed loop complex” via contact with the translation initiation factor eIF4G (Wells et al., 1998; Costello et al., 2015). The RRM2 domain of Pab1p has proved critical both for eIF4G binding during closed loop complex formation and for stimulating translation initiation (Kessler and Sachs, 1998). Intriguingly, the *NIP1* mRNA, while localizing to granules in strains with wild-type *PAB1*, becomes mislocalized in the *PAB1-ΔRRM2* yeast mutant strain (Fig. 4, B and D), but not in strains where other domains of *PAB1* are deleted (data not shown). *NIP1* mRNA is also mislocalized in a *PAB1* double point mutant which carries alterations to two key aromatic residues in RRM1 and RRM2 (*PAB1*-Y83V and F170V). This mutant Pab1p retains the capacity to bind eIF4G but cannot effectively bind poly(A) or promote translation initiation (Kessler and Sachs, 1998). Overall, across a series of *PAB1* mutants either lacking the various domains or carrying key mutations, only those impacting upon translation affect mRNA localization to granules (Fig. 4 B and data not shown). Once again, these results are consistent with mRNA translation being important for localization of translation factor mRNAs to granules in actively growing cells.

### Translation factor mRNAs are likely translated within granules

The majority of granule-associated mRNAs are present as a response to stress (e.g., PBs and SGs) or as part of a finite control of protein expression (e.g., *ASH1* or *bicoid* mRNA localization). As such, these mRNAs enter granules in a translationally repressed state (Besse and Ephrussi, 2008). In contrast, our recent work suggests that two glycolytic mRNAs are actively translated in RNA granules under active growth (Lui et al., 2014). The stem loop insertion and *PAB1* mutant data described above suggest that a similar scenario might exist for the translation factor mRNAs.

In order that a complex and dynamic procedure such as protein synthesis can occur in an RNA granule, the components in the granule would need to be present in a dynamic assembly, such as liquid droplets. A number of nonmembrane-bound compartments have recently been identified to form as a result of liquid–liquid phase separation (Aguzzi and Altmeyer, 2016). The flexible series of fluctuating weak interactions that hold together such droplets make enzymatic activity plausible, whereas it is difficult to envisage such activity within more stably aggregated assemblies (Mitrea and Kriwacki, 2016; Sfakianos et al., 2016). To gain hints as to whether the RNA granules carrying translation factor mRNAs are liquid droplets, 1,6-hexanediol was used. This reagent has been established to disrupt phase-separated liquid droplets while solid particles are unaffected (Kroschwald et al., 2015). Treatment of yeast cells with this reagent led to almost complete disruption of granules bearing the *NIP1* mRNA (Fig. 4, C and D). This reagent also led to the inhibition of translation initiation (Fig. S4), as well as the disruption of other cytoskeletal functions in cells (Wheeler et al., 2016). Whether these effects occur as a result of the general disruption of processes requiring liquid phase particles is currently unknown. Clearly, if sufficient mRNAs are translated in such particles, their disruption would conceivably lead to the translation inhibitory effects observed.

**Figure 5.**
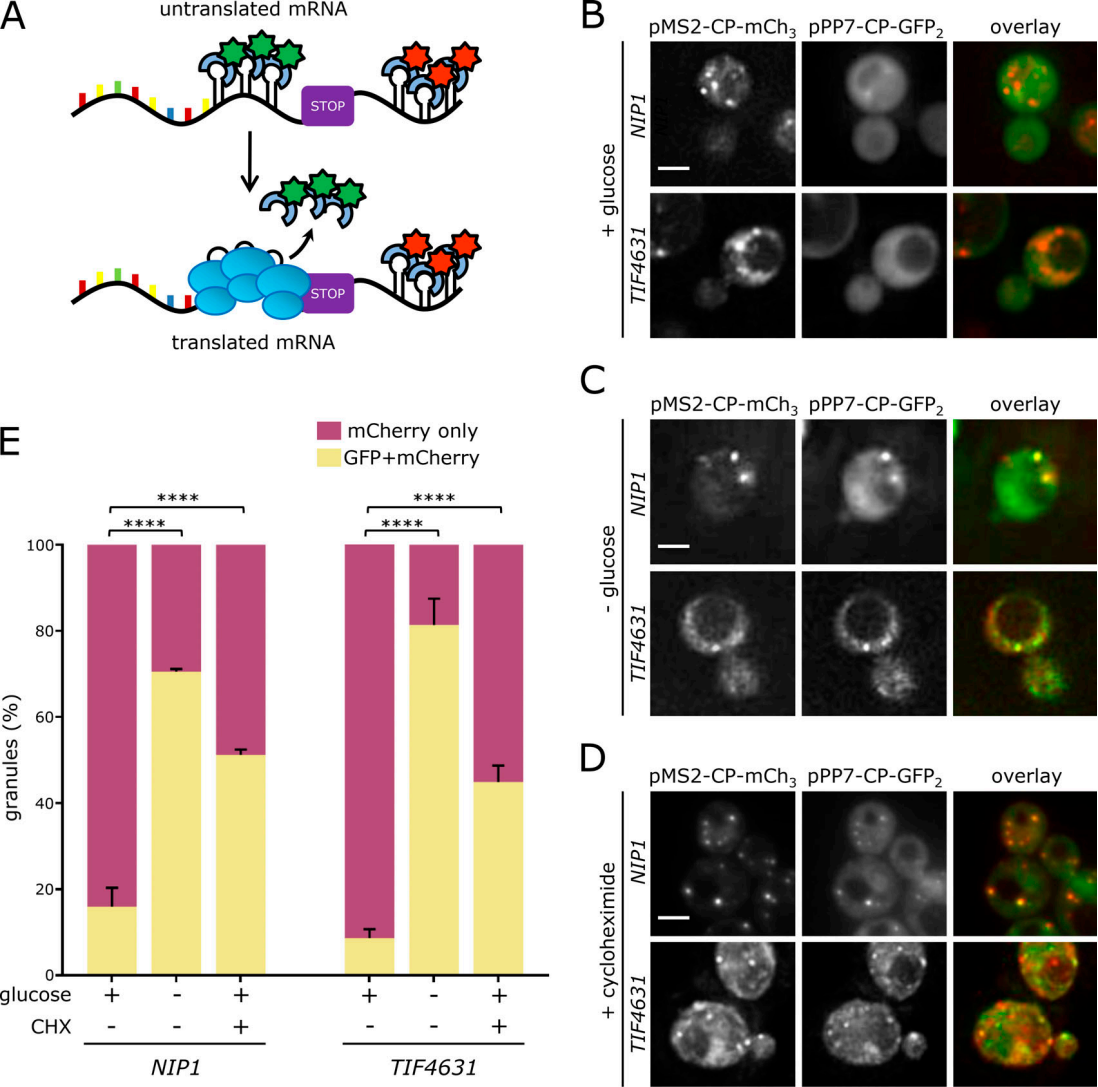
**Granules house translationally active mRNAs. (A)** Schematic for the TRICK strategy. Z-stacked images showing *NIP1*-*TRICK* and *TIF4631*-*TRICK* in rich media (B), after 10 min glucose depletion (C), or in 100 µg/ml cycloheximide (CHX; D). **(E)** Chart evaluating granules showing both GFP and mCherry signal under each condition (*n* = 3, >100 cells per repeat). ****, P < 0.0005. Error bars = +SD. Bars, 4 µm.

To assess whether active translation of translation factor mRNAs can occur within granules, a recently described technique called translating RNA imaging by coat protein knock-off (TRICK; Halstead et al., 2015) was adapted for use in yeast. TRICK relies upon the insertion of *PP7* stem loops within the mRNA coding sequence, upstream of the STOP codon; and *MS2* stem loops downstream of the mRNA STOP codon. If the *TRICK*-tagged mRNA is not translated, the PP7 coat protein fused to GFP and the MS2 coat protein fused to mCherry bind simultaneously, whereas upon translation, the PP7 coat protein is displaced as ribosomes translate the coding region where the PP7 stem loops are sited, resulting in the mRNA only binding the MS2-CP-mCherry (Fig. 5 A).

A *TRICK* tag was precisely inserted into the genome on the *TIF4631* or *NIP1* mRNAs, and the PP7-CP-GFP and the MS2-CP-mCherry fusion proteins were coexpressed. Under active growth conditions, mRNA granules can be observed for both the *NIP1* and *TIF4631* mRNAs in the red but not the green fluorescent channel (Fig. 5, B and F). This suggests that the MS2-CP-mCherry is bound to the mRNAs but that PP7-CP-GFP is not bound (Fig. 5 A). In contrast, after as little as 10 min glucose depletion, which leads to an almost total inhibition of translation initiation (Ashe et al., 2000), both fusion proteins are evident in granules (Fig. 5, C and E). Similarly, cycloheximide treatment, which prevents ribosome translocation, also increases the proportion of granules carrying both fluorescent protein fusions (Fig. 5 D). This result mirrors what has been seen using the TRICK system in mammalian cells (Halstead et al., 2015). It seems likely that the cycloheximide causes decreased ribosomal transit without completely clearing ribosomes from the PP7 stem loop region. Therefore, the level of PP7-GFP fusion protein binding induced by cycloheximide is lower than the level induced by glucose starvation, where ribosomal run-off is particularly extensive relatively to other stress conditions (Holmes et al., 2004).

In sum, these data are highly suggestive that in live cells the translation factor mRNA granules are associated with active translation, and furthermore that this translation is a prerequisite for their localization. This is analogous to our recent studies on mRNA granules housing two glycolytic mRNAs, where we found active protein synthesis was occurring possibly as a means to coregulate protein production (Lui et al., 2014). The localized translation likely occurs in a fluid phase-separated environment, such as has been described in the nucleolus, nuclear pore, and p-granules (Frey et al., 2006; Brangwynne et al., 2009, 2011).

### The translation factor mRNA granules are specifically inherited in a She2p/She3p-dependent manner by the daughter cell

**Figure 6.**
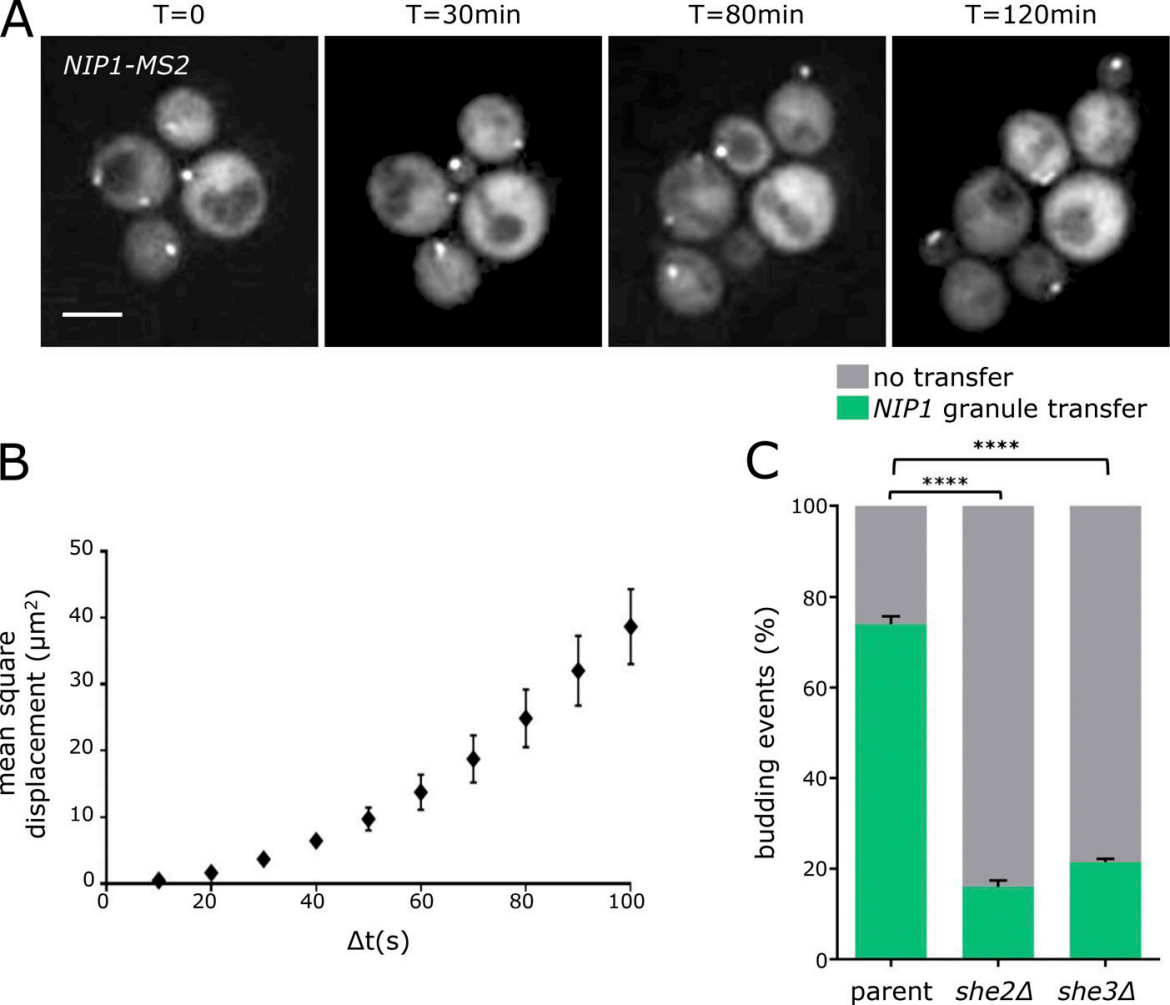
**Granules move to the daughter cell upon division. (A)** Z-stacked images of a *NIP1-MS2* strain imaged over a 2-h period using a microfluidic system. Bar, 4 µm. **(B)** MSD analysis of *NIP1*-*MS2* granules, where displacement over increasing time intervals is evaluated. Error bars = ±SD. **(C)** Chart showing the percentage of budding events where a *NIP1-MS2* granule enters the daughter cell in wild-type, *she2Δ*, and *she3Δ* strains (*n* = 3, >100 cells per repeat). ****, P < 0.0001. Error bars = +SD.

While studying the mRNA localization described above, it became clear that the granules harboring translation factor mRNAs were not evenly inherited during yeast cell division, suggesting that the location of protein production might provide the rationale for the mRNA localization. More specifically, mRNA granules harboring the *NIP1* mRNA were observed to preferentially relocate into the developing daughter cell during the cell cycle (Fig. 6 A). Indeed, across hundreds of cell division events, preferential daughter cell relocalization of *NIP1* mRNA granules is observed in over 70% of cases (Fig. 6 C). Equally, in smFISH studies on endogenous *NIP1* mRNA, roughly 55% of large multi-mRNA granules are found in developing buds, whereas smaller single molecule mRNA foci are significantly less likely to be found at this location (Fig. S5 A).

A well-established method for evaluating particle movement is the mean squared displacement (MSD) analysis (Qian, 2000). The movement of individual *NIP1* mRNA granules was tracked and used to generate MSD plots. In this common analysis (Qian, 2000), the average change in a body’s position, known as the MSD, is plotted over varying time intervals (Δt). The resulting curve provides information about the nature of a body’s movement within cells. Simple Brownian diffusion results in MSD values increasing linearly with Δt (Platani et al., 2002). Such a relationship was not observed in *NIP1* mRNA granule plots: instead, a distinct curve was evident (Fig. 6 B). Similar curves have been associated with a combination of two or more types of movement (Platani et al., 2002; Taylor et al., 2010). For instance, one possible explanation for this curve is that the granules oscillate between mobile and nonmobile phases as a result of binding to transport machinery and a tether, respectively.

The yeast *ASH1* mRNA is well characterized as associated in tethered and mobile states (Gonsalvez et al., 2004). It localizes specifically to the daughter cell as a translationally repressed messenger RNP (mRNP) granule, where it is tethered and translated. The machinery involved in the *ASH1* mRNA movement is particularly well-characterized (Singer-Krüger and Jansen, 2014). She2p is an RNA-binding protein that interacts with the *ASH1* mRNA, and She3p is an intermolecular adaptor connecting She2p to the Myo4p myosin, which travels on actin cables aligned from mother to daughter cell. To evaluate whether the same machinery is involved in the transit of translation factor mRNA granules, the *SHE2* and *SHE3* genes were deleted in strains carrying the *MS2*-tagged *NIP1* mRNA. In the resulting *she2Δ* or *she3Δ* mutants, the level of mRNA granule transfer to daughter cells is dramatically reduced (Fig. 6 C). Even though the machinery is the same as that involved in *ASH1* mRNA transit, *ASH1* and *NIP1* mRNA granules do not colocalize (Fig. S5 B). This observation is consistent with the difference in translational activity of mRNAs housed in these granules, with *ASH1* mRNA being repressed to prevent inappropriate expression during transit, whereas no such repression is evident for the translation factor mRNA granules.

The She2p/She3p machinery has also been implicated in the movement of ER-associated mRNAs (Schmid et al., 2006). It is therefore possible that the translation factor mRNAs are also transported in association with ER. If this were the case, the mRNA granules described above should at least partially overlap with ER. However, no such colocalization of ER and the *NIP1* mRNA granules was discernible (Fig. S5 C), and, in previous datasets (Jan et al., 2014), translation factor mRNAs were not identified as enriched with ER (Fig. S5 D). Similarly, *NIP1* mRNA granules did not appear to colocalize with mitochondria (Fig. S5 E). However, it is still formally possible that the mRNAs are transported in a She2p-dependent manner while very transiently associated with an organelle such as the ER.

Overall, these data support a view that a She2p/She3p-dependent form of mRNA transit is employed in order that the translation factor mRNAs can be preferentially inherited by the daughter cell.

### A switch to filamentous growth is also associated with mRNA granule localization to the developing filamentous daughter cell

Given that a daughter cell will produce its own translation factor mRNAs and the maternal translated protein synthesis machinery is presumably free to diffuse within the cytosol of the mother or the developing daughter cell, it seems highly unlikely that there is an absolute requirement for polarization of translation factor mRNAs into the daughter cell. So why has such a mechanism evolved, and what is the cellular benefit? Energetic considerations suggest that localizing mRNA rather than protein offers a significant advantage. In yeast, each mRNA encodes between 10^2^ and 10^6^ protein molecules, with average estimates in the range of 1,000 to 6,000 protein molecules per mRNA (Futcher et al., 1999; Ghaemmaghami et al., 2003; Lu et al., 2007; Lawless et al., 2016). Clearly, robustly translated mRNAs will generate higher numbers of protein molecules, and localizing such mRNAs versus the several thousand protein molecules they generate offers significant energetic economies. However, in order that this energetic saving is realized, the protein synthetic machinery would also need to be localized to allow translation of the localized mRNAs. Furthermore, the polarization of mRNAs across cells might also relate to potential differing mRNA requirements of the daughter cell relative to the mother. Such a situation might be exacerbated when yeast responds to stress by inducing a different growth program, for example the switch from vegetative to filamentous growth.

**Figure 7.**
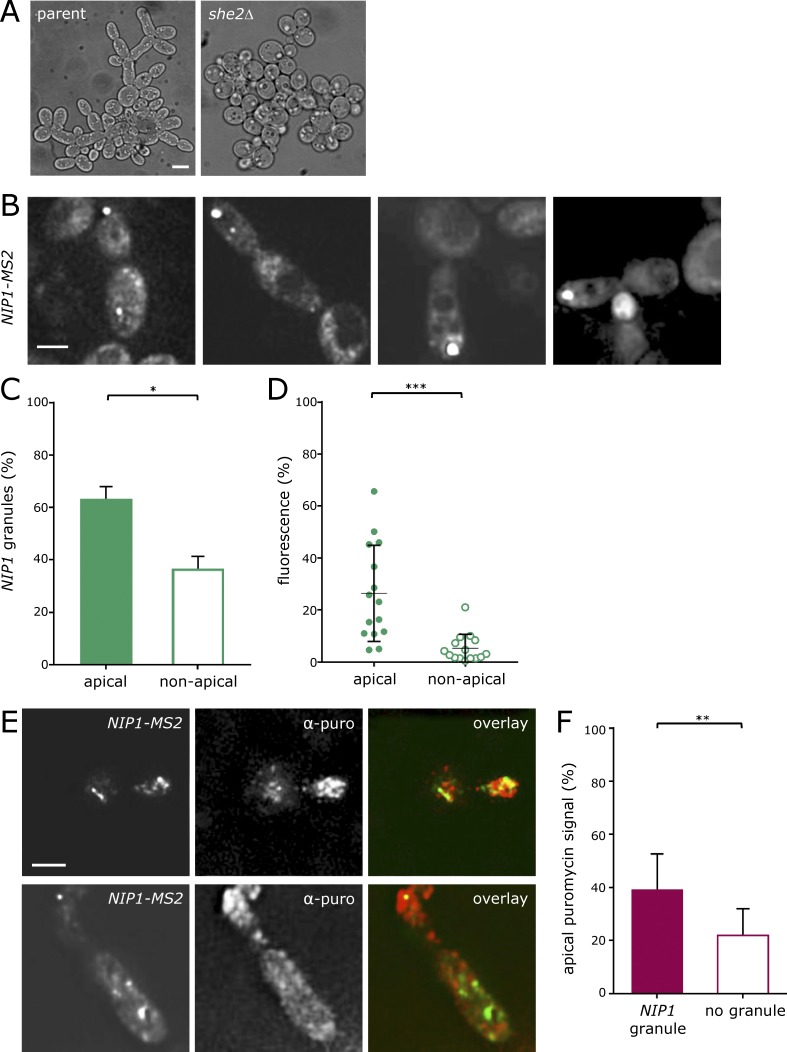
**Granules localizing to the growing ends of cells growing as pseudohyphae. (A)** Images of a *she2Δ* strain relative to a Σ1278b parent after 24 h growth in 1% butanol. **(B)** Z-stacked images of *NIP1-MS2* in a Σ1278b strain showing the filamentous phenotype after 24 h growth in 1% butanol. **(C)** Chart evaluating *NIP1-MS2* granules found in the apical quarter of an elongated cell (*n* = 3, >50 cells per repeat). *, P < 0.05. Error bars = +SD. **(D)** Chart depicting fluorescence in *NIP1* granules from the apical quarter relative to elsewhere. ***, P < 0.0005. Error bars = ±SD. **(E)** Z-stacked images of a Σ1278b strain after a puromycilation assay. Anti-puromycin (red) signal and *NIP1-MS2*/MS2-CP-GFP signal are shown. **(F)** Graph showing the puromycin signal intensity in the apical quarter of elongated cells with or without a colocalizing *NIP1* granule. **, P < 0.01 (*n* = 20 cells). Error bars = +SD. Bars, 4 µm.

Many laboratory strains have lost a capability that is evident in feral yeast to undergo filamentous growth patterns in response to stress conditions (Liu et al., 1996; Lorenz et al., 2000). However, the Σ1278b strain can undergo filamentous growth in response to a range of nutritional stresses including nitrogen limitation, fusel alcohol addition, and glucose depletion (Cullen and Sprague, 2012). Intriguingly, a *she2Δ* mutant in the Σ1278b strain is deficient in the switch from vegetative to filamentous growth and hence fails to undergo this form of polarization (Fig. 7 A). It is entirely possible that a deficiency in the localization of translation factor mRNAs contributes to this phenotype.

To explore the localization of mRNA during the switch to filamentous growth, *NIP1* mRNA granules were followed in Σ1278b strains treated with butanol to induce filamentation (Lorenz et al., 2000): the granules were observed to preferentially localize not only to daughter cells but also to the most apical region of the daughter cell (Fig. 7, B and C). Moreover, the granules found at this position showed on average a higher percentage of total cell fluorescence than granules found elsewhere in the cell (Fig. 7 D), suggesting that a greater proportion of the mRNA localized to this region.

During filamentous growth, following commitment to a new cell cycle, yeast cells continue to grow apically from the growing tip instead of switching to isotropic growth, thus acquiring a characteristic elongated shape (Styles et al., 2013). It seems reasonable that continued apical growth might require a more intense rate of protein production at this site. Indeed, across a range of filamentous fungi, ribosomes or rough ER can be observed in extreme apical regions (Roberson et al., 2010). For instance, a subtending mass of ribosomes has been observed in the spitzenkörper of *Fusarium acuminatu* (Howard, 1981).

To assess whether more robust protein synthetic activity is observable near the apical tip of *Saccharomyces cerevisiae* pseudohyphae, a yeast-adapted ribo-puromycilation assay (David et al., 2011; Lui et al., 2014) was performed on filamentous *S. cerevisiae* cells. In this assay, the addition of cycloheximide prevents polysome run-off so that the translation machinery is locked on the transcript, while puromycin is added to the nascent polypeptide (David et al., 2012). Subsequent immunofluorescence for puromycin allows identification of sites of global translation, while the GFP signal from the *MS2*-tagged mRNA is maintained throughout the procedure. This enables the simultaneous visualization of sites of protein production and *NIP1* mRNA granules. In this analysis, clouds of high puromycin signal were observed to surround prominent mRNA granules (Fig. 7 E). It is important at this point to highlight the earlier result that each granule likely contains a mixture of mRNAs. It is therefore reasonable to assume, when analyzing the localization of *NIP1*, that a number of other translation factor mRNAs might be present in the same location. Interestingly, the percentage of total puromycin signal in the apical quarter of the pseudohyphal cells was measured to be higher in cells carrying a *NIP1* mRNA granule in the same area than in cells showing a granule in other parts of the cell (Fig. 7 F). These data are in accordance with the hypothesis that higher protein production rates are associated with the localization of translation factors to RNA granules.

## Discussion

In this study, we have identified and characterized a previously unanticipated localization for specific mRNAs encoding translation factors. These mRNAs require translation for localization to granules, and the granules themselves appear to represent sites of active translation. Single molecule analysis shows that approximately half of the molecules for each translation factor mRNA are present in large multi-mRNA granules. These large mRNA granules localize specifically to the yeast daughter cell in a mechanism involving the She2p RNA-binding protein. Furthermore, in polarized yeast cells undergoing filamentous growth, the translation factor mRNA granules localize to a region of high protein synthetic activity in the apical region of the elongated daughter cell.

In previous work, we have used an *MS2*-tagging system and FISH to show that the transcript encoding eIF4A (*TIF1*) localizes to granules in growing cells (Lui et al., 2014). Here, we again use the *MS2*-tagging system to show that mRNAs for various other factors involved in translation initiation, elongation, and termination localize to granules. Recent reports have highlighted that caution needs to be applied when interpreting live cell mRNA localization data using MS2-tethering approaches, as it is possible the *MS2* stem loops stabilize mRNA fragments and impact on RNA processing (Garcia and Parker, 2015, 2016; Haimovich et al., 2016; Heinrich et al., 2017). However, it has also been suggested that such phenomena are limited to a subset of transcripts and that such effects are more readily associated with plasmid-based expression systems (Haimovich et al., 2016). From our quantitative RT-PCR (qRT-PCR) data, it appears that the abundance of many of the transcripts analyzed is not affected by insertion of the m-TAG, while for others the tagged version is significantly down-regulated relative to the endogenous version. Given the concerns detailed above and the potential impact of stem loop insertions on mRNA abundance, smFISH analysis was undertaken for endogenous untagged mRNAs. The data obtained accurately reproduce the localization patterns observed with the m-TAG system. In addition, in previous studies, the accumulation of *MS2*-derived mRNA fragments has been shown to coincide with Dcp2p containing foci or PBs (Haimovich et al., 2016). Under the active growth conditions used in our study, PBs are absent: therefore, the RNA granules do not colocalize with Dcp2p or PBs. These data agree with experiments where the insertion of poly(G) stem loops is necessary to observe the accumulation of mRNA 3′ fragments containing *MS2* stem loops under active growth conditions (Sheth and Parker, 2003). Under conditions that induce PBs, such as glucose depletion, colocalization of RNA granules with PBs can however be observed (Lui et al., 2014; Simpson et al., 2014). Similar observations were made here for the translation factor mRNAs, suggesting an involvement of the translation factor mRNA granules in PB formation, where the mRNAs may get degraded as a consequence. Further evidence supporting the validity of the mRNA localization observed in this study stems from the fact that a variety of different transcripts exhibit different patterns of localization even though they all harbor the same *MS2* cassette. Some transcripts are not present in granules, some are present in 20 granules per cell, and translation factor mRNAs are mostly present in fewer than 5 granules per cell. Furthermore, if the *MS2-* and *PP7-*tagging systems in dual-tagged strains were simply detecting mRNA fragments accumulating at sites of degradation, these fragments should all colocalize. However, the data presented here show that the *MS2-* and *PP7*-tagged mRNAs overlap with one another to varying degrees: some overlap completely, some overlap partially, and some do not overlap at all. A final argument supporting the legitimacy of the mRNA localization data presented here comes from the TRICK experiments. These data imply that the mRNAs in the granules are being translated, suggesting that the mRNAs are present in their full form. Therefore, while MS2-tethering strategies can impact upon various aspects of mRNA fate, the approach does allow the investigation of RNA localization in live cells and permits an exploration of the altered localization under changing conditions. FISH approaches allow an investigation of the endogenous mRNA but suffer from a need to fix cells: even if cellular fixation and permeabilization treatments do not lead to alterations in mRNA pattern, they prevent the study of mRNA localization dynamics in live cells.

Similarly to the granules housing glycolytic mRNAs (Lui et al., 2014), the granules carrying translation factors described in this study appear to represent sites of active translation. Furthermore, the capacity of Pab1p to interact with poly(A) tails as well as the translation status of the mRNA seem fundamental for mRNA admittance into these granules. These data are suggestive of a scenario in which translation, or at least the potential for the mRNA to engage in translation, determines the capacity to enter the granule. Given that Pab1p interacts with the polyadenylation machinery, binds mRNA poly(A) tails in the nucleus, and is likely exported with these transcripts (Minvielle-Sebastia et al., 1997; Brune et al., 2005; Dunn et al., 2005), it is possible that certain mRNPs are primed for entry into granules at this early stage. This could potentially offer an explanation as to why, for glycolytic mRNAs and the translation elongation factors mRNAs *TEF1* and *YEF3*, the levels of colocalization within granules mirror similarities in transcription patterns. Indeed, increasing evidence points to inherent connections between the nuclear history of a transcript and its cytosolic fate (Gunkel et al., 1995; Bregman et al., 2011; Trcek et al., 2011; Zid and O’Shea, 2014).

Interestingly, the translational activity of mRNA within the granules is rapidly reversed upon glucose starvation, a condition known to induce PB formation after translation inhibition. In such conditions, the degree of overlap among different mRNAs in the granules increases strikingly, in accordance with the observation that distinct granules coalesce during the formation of PBs (Lui et al., 2014). Considering that yeast PBs have recently been described as liquid-like droplets (Kroschwald et al., 2015) and that the granules described in this work seem to be similarly sensitive to hexanediol treatment, it is not difficult to imagine how the transition from translation granules to PBs could occur, especially given that rapid assembly and exchange of components are facilitated within liquid particles (Kroschwald et al., 2015). One intriguing explanation as to how the granules coalesce when forming PBs is that a glucose starvation–induced “contraction” of the cytosol (Joyner et al., 2016) might induce fusion of the granules by simple molecular crowding effects, or as a consequence of an altered phase separation between the granules and the cytosol.

What emerges from these observations is a scenario in which certain mRNAs exist in RNP granules, where they can either undergo translation or decay, depending on cellular requirements. A role for RNA-containing granules in mRNA degradation, storage, or localization is widely reported, where such granules are generally associated with translation repression, while the potential for specialized translation foci is less widely acknowledged. One advantage in colocalizing mRNAs to translation foci is the potential for cotranslational assembly of protein–protein complexes (Shiber et al., 2018). Indeed, many of the translation initiation factors are present as complex multi-subunit factors. For example, we have investigated the localization of mRNAs encoding components of eIF2B, eIF2, and eIF3, and it is possible that these complexes are constructed cotranslationally.

In recent years, there is an increased appreciation of a potential relationship between mRNA colocalization and protein complex formation. In yeast, from a study of 12 multi-subunit protein complexes, 9 were shown to form cotranslationally (Shiber et al., 2018). Likewise, in human cells, the dynein heavy chain mRNA colocalizes at translation sites, possibly as a way to facilitate protein complex assembly (Pichon et al., 2016). Similarly, mRNAs for many of the components of the Arp2/Arp3 complex are localized and cotranslated at the leading edge of fibroblasts, possibly to aid in protein complex formation (Mingle et al., 2005; Willett et al., 2013). Equally, the peripherin mRNA localizes to specialized factories that couple the translation of the transcript with the assembly of peripherin intermediate filaments in a process termed dynamic cotranslation (Chang et al., 2006).

A key feature of all these examples is the necessity for specific mRNA translation in distinct cellular regions and hence the presence of the translation machinery at this locale. A concentration of the translation machinery in certain areas of cells has previously been associated with asymmetric growth: in migrating fibroblasts, translation factors can preferentially localize to lamellipodia, where rates of protein production are higher (Willett et al., 2010, 2011). Furthermore, local translation is a key regulator of cellular protrusions in migrating mesenchymal cells (Mardakheh et al., 2015).

In this study, we show that translation factor mRNA granules are transported to the daughter cell in a She2p/She3p-dependent manner. This localization of translation factor mRNAs provides a compelling rationale for the RNA granules, as they might provide the daughter cell with a “start-up” pack concentrating protein synthetic activity to facilitate daughter cell development. Given that approximately half the molecules of each individual mRNA are present in such granules, a mother cell is essentially donating half of the mRNA to the developing daughter. Such an idea has parallels with maternal mRNA inheritance in oocytes for organisms such as *Xenopus* and *Drosophila* (Lee et al., 2014). We propose that the granules represent specialized factories for the translation machinery, which are specifically inherited by the daughter cell. As such, protein synthetic activity would be concentrated in an area of the cell where it is particularly required.

## Materials and methods

### Strains and plasmids

The *S. cerevisiae* strains used in this study are listed in Table S1. *MS2* and *PP7* stem loops were amplified by PCR from the pLOXHIS5MS2L and pDZ416 plasmids, respectively, using primers directed to the 3′ UTR of the relevant genes. After transformation and selection, accurate homologous recombination of the resulting cassette was verified using PCR strategies, and the selection marker was subsequently excised using Cre recombinase. pMS2-CP-GFP_3_, pMS2-CP-mCherry_3_, or pMet25MCP-2yEGFP (pDZ276) plasmids were then transformed into the strains to enable detection of *MS2-* and *PP7*-tagged mRNAs. The *MS2* and *PP7* tagging reagents were gifts from J. Gerst (Weizmann Institute of Science, Rehovot, Israel) and R. Singer (Albert Einstein College of Medicine, New York, NY; Addgene 31864 and 35194; Haim-Vilmovsky and Gerst, 2009; Hocine et al., 2013). Dual *MS2-* and *PP7*-tagged strains were obtained by mating of appropriate haploid strains, followed by sporulation and tetrad dissection. TRICK strains were generated using a similar approach, but using a DNA template developed for TRICK in yeast. Briefly, a 12x*PP7* 24x*MS2* synthesized fragment (Halstead et al., 2015) was subcloned into the pFA6a-kanMX6 vector, and specific targeting primers were used to isolate the TRICK region with the marker gene such that integration into the *NIP1* and *TIF4631* genes was achieved. For *she2Δ* and *she3Δ* strains, the ORFs were replaced by the nourseothricin resistance gene (*natNT2*) amplified from the pZC2 vector (Carter and Delneri, 2010). A *PAB1* shuffle strain was generated in the yMK2254 *NIP1-MS2* strain by first transforming a *PAB1 URA3* plasmid, then deleting the *PAB1* gene with a *LEU2* cassette. *PAB1* mutant strains were generated by transformation of *PAB1-ΔRRM2 TRP1* and *PAB1-Y83V,F170V TRP1* plasmids (Kessler and Sachs, 1998) into the shuffle strain followed by expulsion of the *PAB1 URA3* plasmid. For generation of the yEPlac195-*NIP1* plasmid, *MS2*-tagged *NIP1* was amplified from the yeast strain yMK2254 and cloned into yEPlac195 (Gietz and Sugino, 1988). A stem loop sequence (Vattem and Wek, 2004) was inserted into this plasmid using a PCR-based approach, where the stem loop was introduced on primers that directed amplification of the entire plasmid, which was subsequently verified by DNA sequencing.

### Yeast growth

Strains were grown at 30°C on Synthetic Complete medium with 2% glucose (SCD) with selection where necessary (Sherman, 1991). Cells were incubated for 30 min in SCD media lacking methionine to induce expression the coat protein–GFP/RFP fusions before imaging. For experiments requiring glucose starvation, exponentially growing cells were resuspended in media lacking glucose, then incubated for 10 min at 30°C before imaging. For induction of filamentous growth, the JCY100 strain (Σ1278b background; Cook et al., 1997) was grown in SCD media containing 1% butanol for up to 24 h at 30°C before imaging.

### Fluorescent microscopy

Live cell microscopy was performed on a Delta Vision microscope (Applied Precision) equipped with a Coolsnap HQ camera (Photometrics), using a 100×/1.40 NA oil plan Apochromat objective. Imaging was performed for GFP (excitation, 490/20 nm; emission, 535/50 nm; exposure, 200–400 ms), mCherry (excitation, 572/35 nm; emission, 632/60 nm; exposure, 400–800 ms), and CFP (excitation, 436/10 nm; emission, 465/30; exposure, 600–800 ms). Images were acquired using Softworx 1.1 software (Applied Precision) and processed using the ImageJ software package (National Institutes of Health). For routine live-cell imaging, exponential yeast were viewed on poly-L-lysine–coated glass slides at room temperature. For live-cell imaging over longer periods of time, a microfluidic system (CellASIC; Merck Millipore) was used, where exponential yeast were imaged every 10 min for 2 h at 30°C. For smFISH, images of fixed samples mounted in ProLong^T^ diamond antifade mountant with DAPI (Life Technologies) were collected at room temperature on a Leica TCS SP8 AOBS inverted gSTED microscope using a 100×/1.40 NA Plan Apochromat objective and 1× confocal zoom, with LAS X software (Leica). The confocal settings were as follows: pinhole 1 airy unit, scan speed 400 Hz bidirectional, format 1,984 × 1984. DAPI images were collected using a photon multiplying tube detector, with a blue diode 405-nm laser (5%). Confocal images were collected using hybrid detectors with the following detection mirror settings; Alexa Fluor 488, 410–483 nm (5–50 µs gating); Alexa Fluor 546, 556–637 nm (5–35 µs gating); and Alexa Fluor 647, 657–765 nm (5–50 µs gating) using the 488-nm (60%), 546-nm (60%), and 646-nm (60%) excitation laser lines, respectively. Images were collected sequentially in 200-nm Z sections. Acquired images were subsequently deconvolved and background subtracted using Huygens Professional (Scientific Volume Imaging). Maximum projections of these images were generated using FIJI.

### smFISH and immunofluorescence

For smFISH, gene-specific 20-nt antisense oligonucleotides were designed with a 5′ Flap sequence, to which fluorescently labeled oligonucleotides were annealed (Tsanov et al., 2016). 30–48 probes were designed per mRNA such that each probe had minimal potential for crosshybridization and between 40 and 65% guanine-cytosine content. To generate the fluorescently labeled smFISH probes, 200 pmol of an equimolar mix of gene-specific oligos was annealed with 250 pmol of the appropriate fluorescently labeled flap oligo (Y-Flap-Alexa Fluor 488, X-Flap-Alexa Fluor 546, and Z-Flap-Alexa Fluor 647; Integrated DNA Technologies) in 1× NEBuffer 3 (New England Biolabs; Tsanov et al., 2016). To perform smFISH, strains were grown in SCD overnight to mid-log phase and fixed with 4% EM-grade formaldehyde (15714-S; Electron Microscopy Sciences) for 45 min, at room temperature. After fixation, cells were washed with buffer B (1.2 M sorbitol and 100 mM KHPO_4_, pH 7.5), then resuspended in spheroplasting buffer (1.2 M sorbitol, 100 mM KHPO 4, 20 mM Ribonucleoside Vanadyl Complex, 0.2% β-mercaptoethanol, and 1 mg/ml lyticase) and incubated at 37°C for 15 min before being permeabilized with 70% ethanol. Subsequently, cells were hybridized with 20 pmol of the appropriate fluorescently labeled smFISH probes in hybridization buffer (10 mg *E. coli* tRNA, 2 mM Ribonucleoside Vanadyl Complex, 200 µg/ml BSA, 10% dextran sulfate, 10% formamide, and 2× SSC in nuclease-free water). Cells were then washed in 10% formamide and 2× SSC and adhered to 0.01% poly-L-lysine–coated coverslips before mounting in ProLong^T^ diamond antifade mountant with DAPI (Life Technologies).

For immunofluorescence, cells were grown to mid-log phase in media with 1 M sorbitol, incubated for 1 h with 1 mg/ml lyticase, and then incubated for 20 min with 1 mg/ml puromycin and 100 µg/ml cycloheximide. Cells were then fixed in 4% formaldehyde and loaded on poly-L-lysine–coated coverslips. Coverslips were blocked for 30 min in 4% BSA, then incubated overnight with a mouse anti-puromycin monoclonal antibody (1:1,000 in 4% BSA; Millipore). After a 1× PBS wash, coverslips were incubated with an anti-mouse Texas red–conjugated secondary antibody (1:200 in 4% BSA; Abcam) for 2 h, and then mounted and imaged.

### Quantification and statistics

For quantification of granule numbers per cell, 100 cells were counted for each strain over three biological repeats. For quantification of overlapping MS2 and PP7 signal in double-tagged strains or TRICK strains, 100–150 granules were considered for each strain over three biological repeats. For quantification of budding events and the inheritance of granules, all the budding events observable (∼30) over three different frames were considered for each strain over three biological repeats. For quantification of granules found in the apical quarter of filamentous cells, the length of the cell was calculated using ImageJ, and granules found within a quarter of the length from the apical end were counted. Three biological repeats were considered, with at least 150 cells counted per repeat. For quantification of percentage of fluorescence, the intensity of fluorescence was measured using ImageJ for 20 cells. The corrected total fluorescent intensity for the whole cell and for the granules was measured to calculate the percentage of fluorescence in granules. GraphPad Prism 7 (GraphPad Software) was used to produce the graphs and to calculate the SEM, indicated by error bars. Two-way ANOVA was performed using GraphPad Prism 7.

smFISH micrographs were analyzed using FISHQuant (Mueller et al., 2013) and FindFoci (Herbert et al., 2014) to provide sub-pixel resolution of spot locale and spot enhancement via dual Gaussian filtering. The resulting output files were then processed using custom scripts in R to assess spot colocalization, mRNA copies per spot, and mRNA copies per cell. For spot colocalization analysis, each spot in one channel was paired with the closest spot in the opposite channel based on spot centroid distance in 3D space. Spots were deemed to colocalize if the 3D distance between them was less than the summed radius of the two spots. To assess the number of mRNAs in each spot, the cumulative fluorescent intensity of all spots was calculated and fit to a Gaussian curve, the peak of which corresponds to the intensity of a spot containing a single mRNA (Trcek et al., 2017). This value was used to normalize the cumulative intensity of each spot, thus determining the number of mRNAs per spot (Trcek et al., 2012). Subsequently, the mean number of mRNAs per cell was calculated using these values and cross-compared with values obtained from genomic studies using RNA-seq (Lawless et al., 2016; Lahtvee et al., 2017). For micrograph pseudo-coloring, foci were assigned a grayscale value corresponding to the number of predicted mRNAs within that spot, calculated as above. Subsequently, these grayscale intensities were “colored” and visualized using a custom LookUp Table.

### MSD

Strain yMK2254 was imaged at intervals of 10 s over a total time of 2 min. Granules were followed, and the distance moved was measured using ImageJ. The distances traveled by granule in 10-s intervals were used to calculate the MSD using the equation MSD (Δt) = [d(t) − d(t + Δt)]^2^, where Δt is the time interval between images and d(t) is the position of the RNA granule at a given time t (Platani et al., 2002).

### qRT-PCR

To extract RNA, 50 ml mid-log phase yeast cultures were pelleted and resupended in 1 ml Trizol (Thermo Fisher Scientific), and then 400 µl acid-washed beads (Sigma-Aldrich) were added. Tubes were sequentially vortexed five times for 20 s with 1-min intervals. 150 µl chloroform was added, and the samples were mixed. The tubes were centrifuged in a microfuge for 15 min at 12, 000 × g. The aqueous layer was collected, and 350 µl isopropanol was added. The resulting precipitate was collected via centrifugation in a microfuge for 15 min at 12,000 × g and washed in 75% ethanol. The resulting pellet was resuspended in 20 µl of nuclease-free H_2_O. qRT-PCR was performed using 300 ng RNA with the CFx Connect Real-Time system with the iTaq Universal SYBR Green One Step Kit (Bio-Rad) according to the manufacturer’s instructions. Primers were designed to amplify a 200-nt region just upstream of the STOP codon. Samples were run in triplicates and normalized to *ACT1* mRNA, and the fold change was calculated using 2^−ΔCt^ for each tested RNA.

### Online supplemental material

Fig. S1 summarizes the effects of the insertion of the MS2 tag on the target mRNA. Fig. S2 shows controls and smFISH validation for the colocalization data shown in Fig. 2. Fig. S3 compares the localization of genome-based and plasmid-based NIP1-MS2. Fig. S4 shows the effect of 1,6-hexanediol on translation initiation. Fig. S5 shows that NIP1 does not colocalize with other asymmetrically inherited mRNAs or organelles. Table S1 lists the yeast strains used in this study.

## Supplementary Material

Supplemental Materials (PDF)
