# Supplementary material for: Translation factor mRNA granules direct protein synthetic capacity to regions of polarized growth
Source: J Cell Biol. 2019 Mar 15;218(5):1564–81. doi: 10.1083/jcb.201704019 (PMC6504908; doi:10.1083/jcb.201704019)
Supplement: Supplemental Materials (PDF) [file JCB_201704019_sm.pdf]

Supplemental material

Pizzinga et al., https://doi.org/10.1083/jcb.201704019

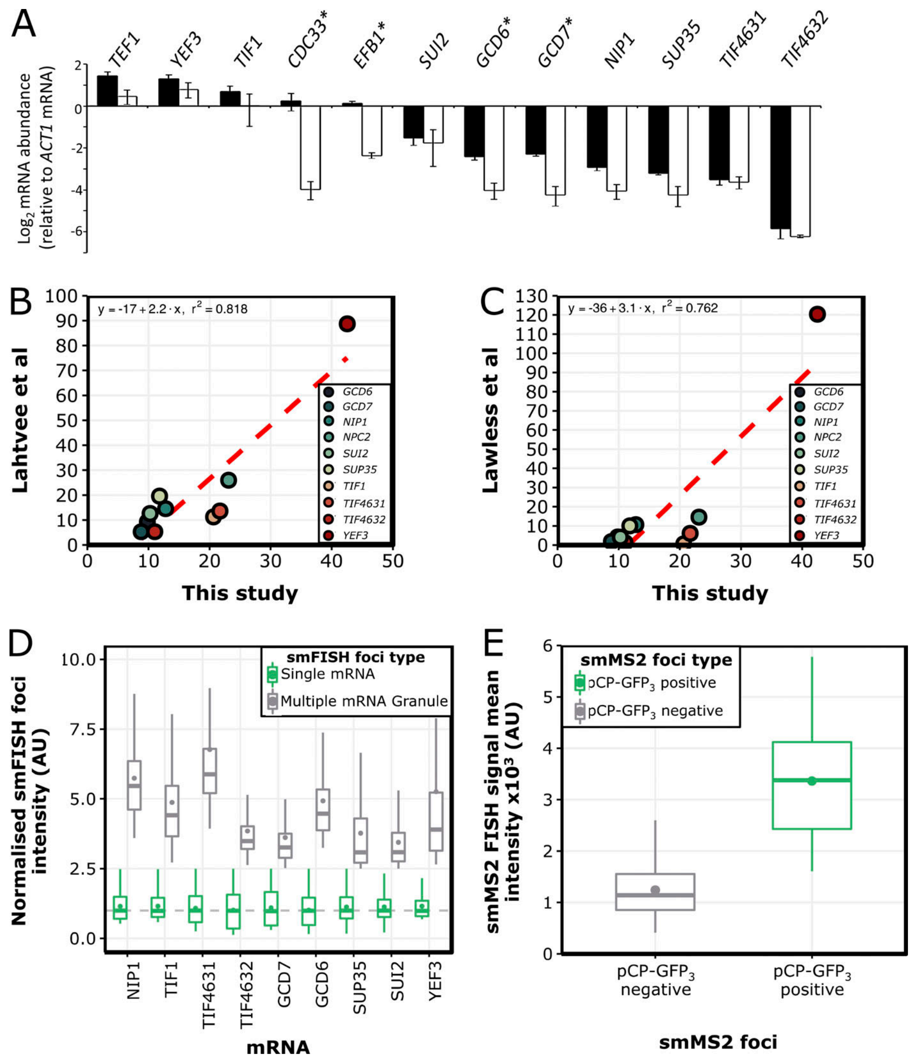

Figure S1. **The MS2 system used has variable effects on mRNA abundance, yet accurately reports on multi-mRNA granules. (A)** Translation factor mRNA abundances quantified relative to *ACT1* mRNA for endogenous mRNAs (parent strain; black bars) or MS2-tagged mRNA (m-TAG strain; white bars). Asterisks denote mRNAs where stem loop introduction significantly altered mRNA level ($P < 0.05$). Error bars = ±SD. **(B and C)** Scatterplots comparing mRNA copies per cell as calculated in this study using smFISH and in two recently published studies using RNA-seq approaches (Lawless et al., 2016; Lahtvee et al., 2017). The dashed red line is the linear regression line, defined by the equation. R^2 indicates the coefficient of determination ($n > 1,000$ foci per mRNA). **(D)** Boxplot showing the difference in smFISH intensities for small mRNA granules versus large across the panel of mRNAs studied. Boxplots are colored depending on smFISH foci size: either single mRNA, with <2.5 mRNAs/foci (green), or multiple mRNA granules, with >2.5 mRNAs/foci (gray). Circles depict the mean, and the dashed line depicts y = 1 ($n > 1,000$ foci per mRNA). **(E)** Boxplot for the difference in smFISH signal intensity for granules where the MS2-CP–GFP signal is evident (green), versus those where this signal is absent (gray; $n = 4,429$ foci). Circles represent the mean intensity value.

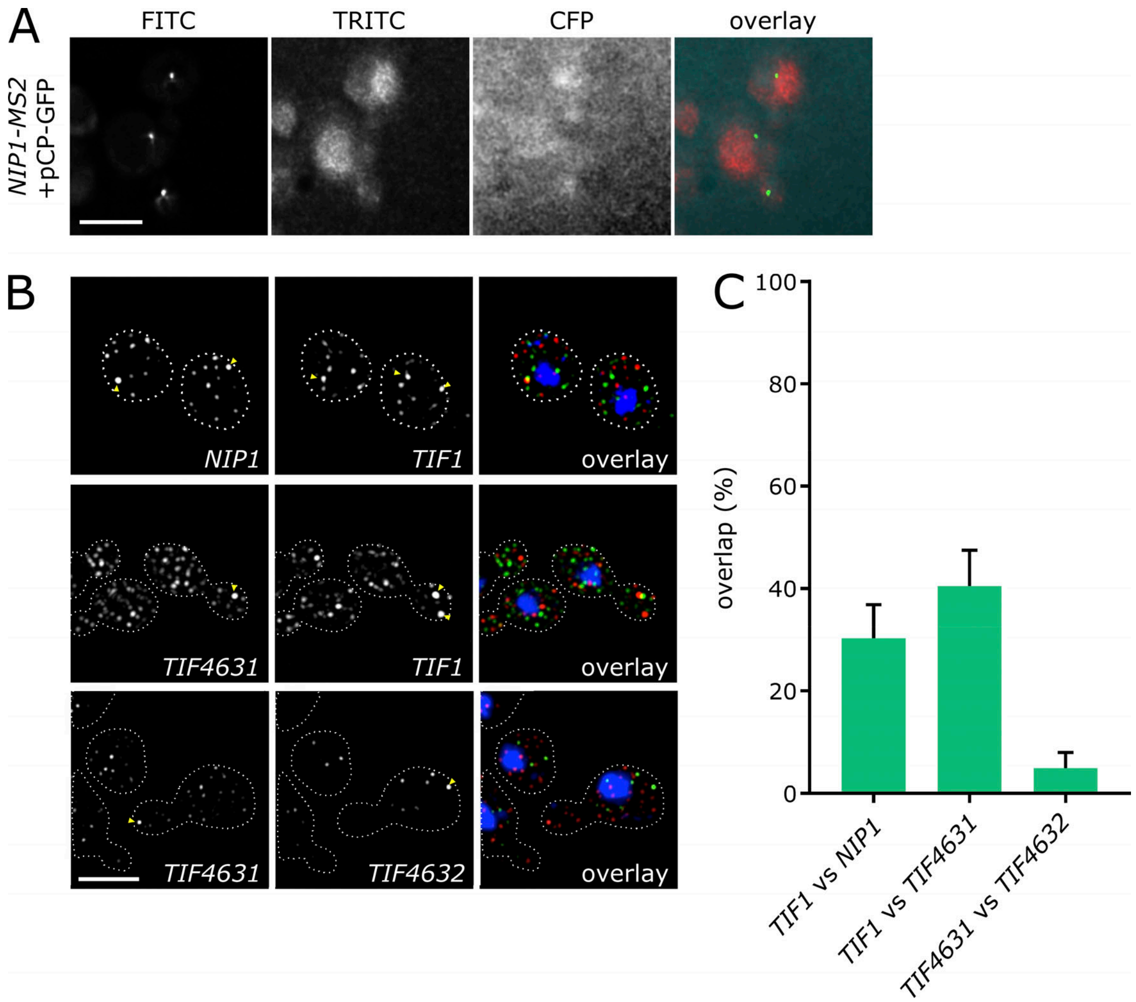

Figure S2. **Colocalization is not a result of fluorescent channel crosstalk and can be verified by smFISH. (A)** Z-stacked images showing pCP-GFP signal, attributed to *NIP1-MS2,* acquired in FITC, TRITC, and CFP. TRITC and CFP channels were acquired at long exposure (1 s) to assess channel crosstalk. FITC was acquired for 100 ms. Bar, 5 µm. **(B)** Z-stacked images showing localization of endogenous *TIF1* versus *NIP1* mRNAs, *TIF1* versus *TIF4631* mRNAs, and *TIF4631* versus *TIF4632* mRNAs. Yellow arrows indicate multi-mRNA–containing foci. **(C)** Chart shows the percentage of observable *TIF1*, *NIP1*, *TIF4631*, and *TIF4632* mRNA granules that colocalize, as indicated (n > 100 cells). Error bars = +SD. Bar, 3 µm.

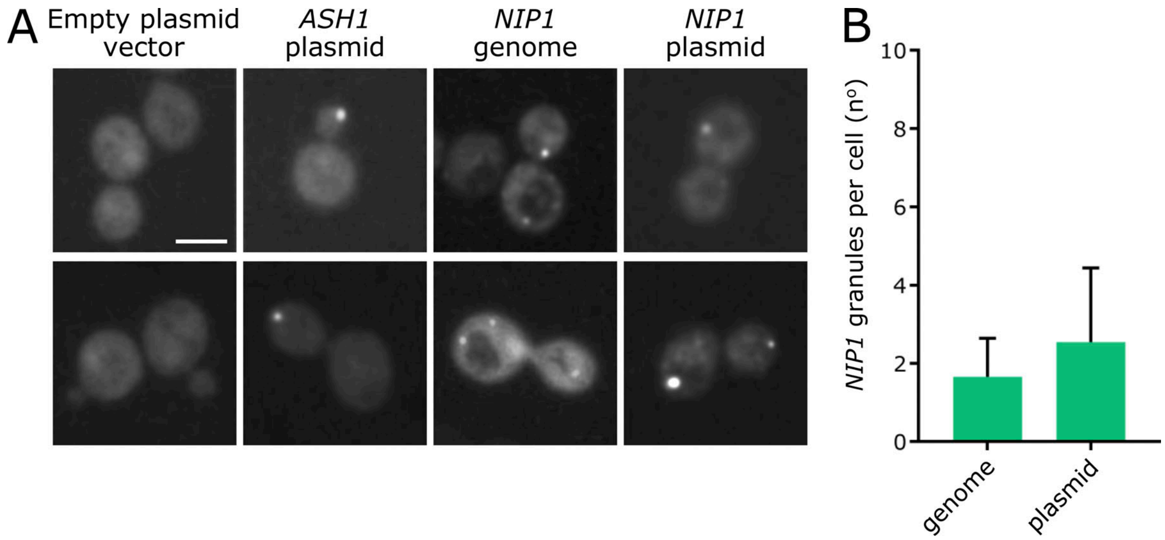

Figure S3. **A comparison of the localization of plasmid-derived *NIP1-MS2* mRNA and *NIP1-MS2* from the genome. (A)** Z-stacked fluorescent microscopy images of yeast strains expressing MS2-CP-GFP and bearing either the empty plasmid, an *ASH1-MS2* control plasmid, a *NIP1-MS2* plasmid, or the genome version of *NIP1-MS2*. Two images are shown for each strain. Bar, 5 µm. **(B)** Chart showing the number of granules per cell in the genome and plasmid-based *NIP1* tagged strains. 50 cells were considered. Error bars = +SD.

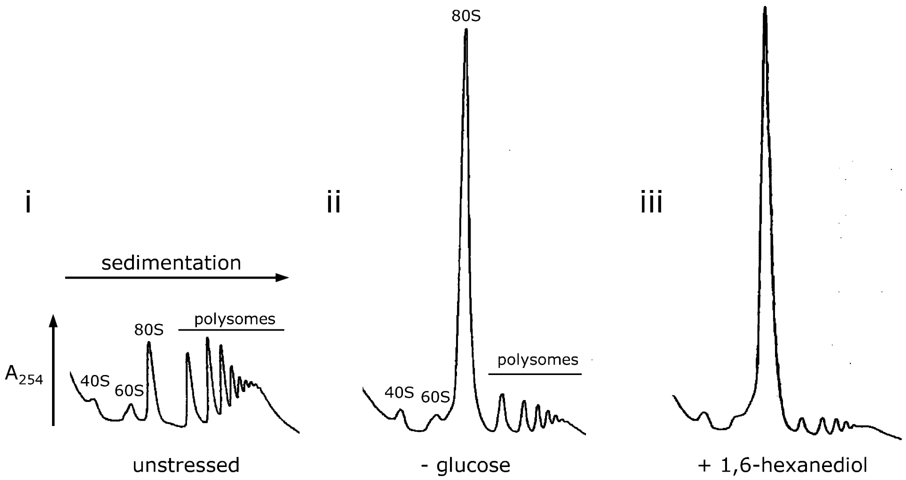

Figure S4. **1,6-hexanediol inhibits translation initiation.** Polysome traces from the yMK1647 strain grown in synthetic complete media (SCD) then transferred to (i) control SCD media, (ii) media lacking glucose for 10 min, and (iii) SCD media with 10% 1,6-hexanediol for 30 min. Polysomes were analyzed as described in the Materials and methods. The 40S (small ribosomal subunit), 60S (large ribosomal subunit), 80S (monosome), and polysome peaks are labeled, as well as the direction of sedimentation and the A_{254} axis.

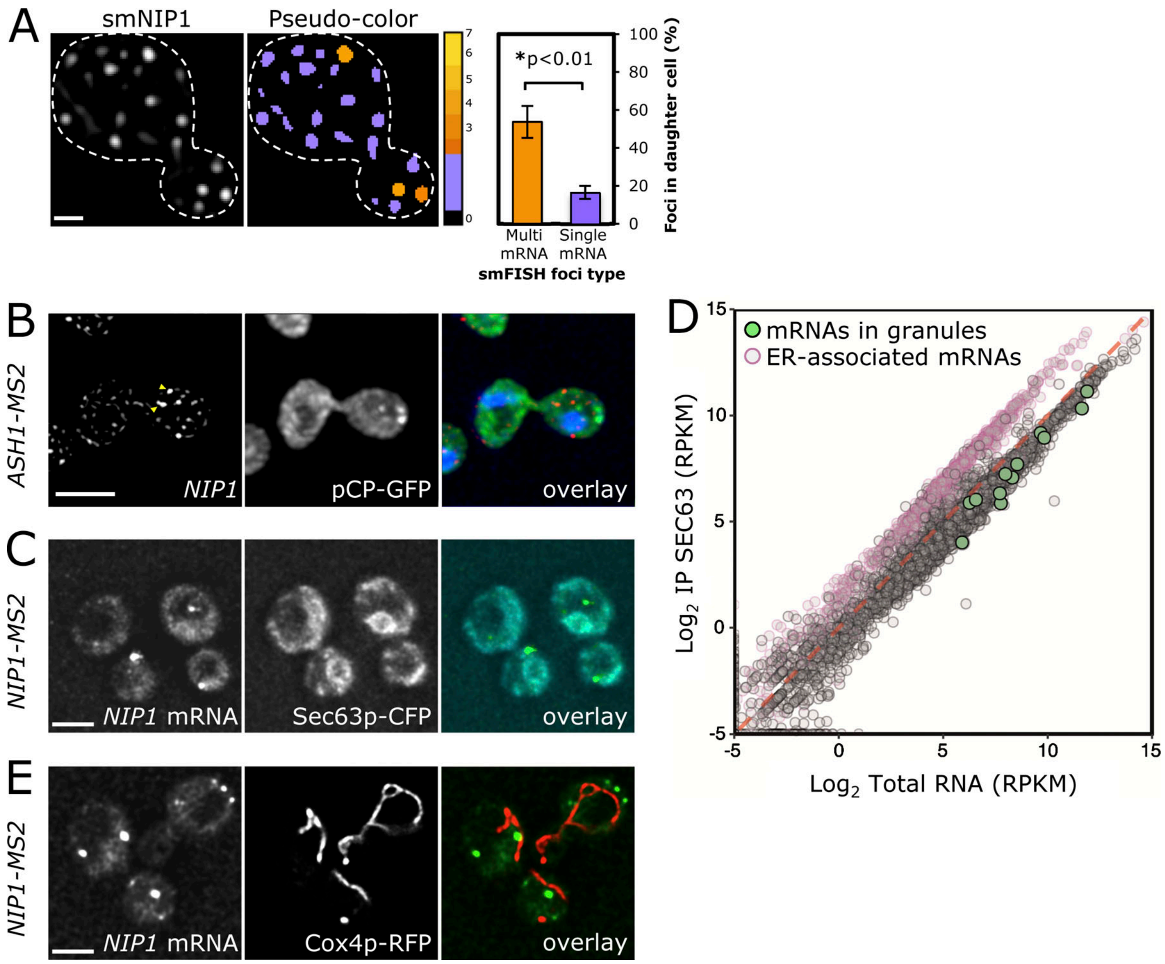

Figure S5. **Translation factor mRNA granules are asymmetrically inherited, but do not colocalize with other asymmetrically inherited organelles or mRNAs. (A)** Z-stacked fluorescent microscopy images of a fixed untagged yeast strain probed for *NIP1* mRNA localization using smFISH. Pseudo-colored image showing predicted mRNA per foci based on background-subtracted cumulative intensity (see Materials and methods). Multi-mRNA foci are classified as foci containing >2.5 mRNAs. Statistical significance determined using Welch's two-sample *t* test. Bar, 1 µm. **(B)** Z-stacked fluorescent microscopy images of a fixed yeast strain expressing MS2-tagged *ASH1* and the MS2 coat protein GFP fusion, probed for *NIP1* mRNA localization using smFISH. **(C)** Z-stacked fluorescent microscopy images of a yeast strain expressing MS2-tagged *NIP1* and the MS2 coat protein GFP fusion, as well as the ER marker Sec63p-CFP. **(D)** A scatterplot detailing ER-associated mRNAs and non–ER-associated mRNAs (data from Jan et al., 2014) as defined by the degree of enrichment with Sec63. The translation factor mRNAs identified in granules are depicted in green and do not overlap with the ER-associated mRNAs. RPKM, reads per kilobase of transcript per million mapped reads. **(E)** Z-stacked fluorescent microscopy images of a yeast strain expressing MS2-tagged *NIP1* and the MS2 coat protein GFP fusion, as well as the mitochondrial marker Cox4p, tagged with RFP. Bars, 3 µm.

Table S1. **Yeast strains used in this study**

Strain	Genotype	Source
yMK7	*MATa leu2Δ::hisG his3Δ::hisG trp1Δ::hisG ura3-52*	JCY100 (J. Thorner)
yMK467	*MATα ADE2 his3-11,15 leu2-3,112 trp1-1 ura3-1*	Ashe strain collection
yMK807	*MATa ADE2 his3-11,15 leu2-3,112 trp1-1 ura3-1*	Ashe strain collection
yMK1585	yMK467 *TIF1-MS2L p[MS2-GFP$_3$ HIS3]*	Ashe strain collection
yMK1741	yMK467 *p[MS2-GFP$_3$ HIS3]*	Ashe strain collection
yMK1833	yMK467 *CDC33-RFP::NAT DCP2-CFP::TRP1 NPC2-MS2L p[MS2-GFP$_3$ HIS3]*	Ashe strain collection
yMK2124	yMK467 *SUP35-MS2L p[MS2-GFP$_3$ HIS3]*	This study
yMK2134	yMK467 *GCD6-MS2L p[MS2-GFP$_3$ HIS3]*	This study
yMK2136	yMK467 *GCD7-MS2L p[MS2-GFP$_3$ HIS3]*	This study
yMK2218	yMK466 *TIF1-PP7L p[PP7-GFP$_2$ URA3]*	This study
yMK2249	yMK467 *CDC33-MS2L p[MS2-GFP$_3$ HIS3]*	This study
yMK2251	yMK466 *ENO2-PP7L p[PP7-GFP$_2$ URA3]*	This study
yMK2254	yMK467 *NIP1-MS2L p[MS2-GFP$_3$ HIS3]*	This study
yMK2272	yMK467 *TIF4632-MS2L p[MS2-GFP$_3$ HIS3]*	This study
yMK2362	yMK467 *EFB1-MS2L p[MS2-GFP$_3$ HIS3]*	This study
yMK2363	yMK467 *YEF3-MS2L p[MS2-GFP$_3$ HIS3]*	This study
yMK2364	yMK467 *TIF4631-MS2L p[MS2-GFP$_3$ HIS3]*	This study
yMK2365	yMK466 *NIP1-MS2L TIF1-PP7L p[MS2-mCh$_3$ HIS3] p[PP7-GFP$_2$ URA3]*	This study
yMK2369	yMK467 *she2::NAT NIP1-MS2L p[MS2-GFP$_3$ HIS3]*	This study
yMK2370	yMK467 *she3::NAT NIP1-MS2L p[MS2-GFP$_3$ HIS3]*	This study
yMK2372	yMK467 *TIF4631-MS2L TIF1-PP7L p[MS2-mCh$_3$ HIS3] p[PP7-GFP$_2$ URA3]*	This study
yMK2373	yMK467 *EFB1-MS2L TIF1-PP7L p[MS2-mCh$_3$ HIS3] p[PP7-GFP$_2$ URA3]*	This study
yMK2519	yMK467 *TEF1-MS2L p[MS2-GFP$_3$ HIS3]*	This study
yMK2542	yMK467 *TIF4632-PP7L p[PP7-GFP$_2$ URA3]*	This study
yMK2564	yMK7 *p[NIP1-MS2L] p[MS2-GFP$_3$ HIS3]*	This study
yMK2567	yMK467 *pab1::LEU2 NIP1-MS2L p[MS2-GFP$_3$ HIS3] p[PAB1 TRP1]*	This study
yMK2614	yMK467 *SUI2-MS2L p[MS2-GFP$_3$ HIS3]*	This study
yMK2616	yMK467 *pab1::LEU2 NIP1-MS2L p[MS2-GFP$_3$ HIS3] p[PAB1-ΔRRM2 TRP1]*	This study
yMK2617	yMK467 *pab1::LEU2 NIP1-MS2L p[MS2-GFP$_3$ HIS3] p[PAB1-Y83V,F170V TRP1]*	This study
yMK2672	yMK467 *TEF1-MS2L ENO2-PP7L p[MS2-GFP$_3$ HIS3] p[PP7-GFP$_2$ URA3]*	This study
yMK2686	yMK467 *TIF4631-TRICK-stop-MS2L p[MS2-mCh$_3$ HIS3] p[PP7-GFP$_2$ URA3]*	This study
yMK2687	yMK467 *DCP2-CFP::TRP1 NIP1-MS2L TIF1-PP7L p[MS2-mCh$_3$ HIS3] p[PP7-GFP$_2$ URA3]*	This study
yMK2688	yMK467 *NIP1-TRICK-stop-MS2L p[MS2-mCh$_3$ HIS3] p[PP7-GFP$_2$ URA3]*	This study
yMK2741	yMK7 *she2::NAT*	This study
yMK2941	yMK467 *p[NIP1-MS2 URA3] p[MS2-GFP$_3$ HIS3]*	This study
yMK2942	yMK467 *p[sl-NIP1-MS2 URA3] p[MS2-GFP$_3$ HIS3]*	This study
yMK2949	yMK467 *TIF4631-MS2L TIF4632-PP7L p[MS2-mCh$_3$ HIS3] p[PP7-GFP$_2$ URA3]*	This study
yMK3076	yMK467 *NIP1-MS2L SEC63-CFP::TRP1 p[MS2-GFP$_3$ HIS3]*	This study
yMK3083	yMK467 *NIP1-MS2L p[MS2-GFP$_3$ HIS3] p[COX4-RFP URA3]*	This study
yMK3219	yMK467 *ASH1-MS2L p[MS2-GFP$_3$ HIS3]*	This study

References

Jan, C.H., C.C. Williams, and J.S. Weissman. 2014. Principles of ER cotranslational translocation revealed by proximity-specific ribosome profiling. *Science.* 346: 1257521. https://doi.org/10.1126/science.1257521

Lahtvee, P.J., B.J. Sanchez, A. Smialowska, S. Kasvandik, I.E. Elsemman, F. Gatto, and J. Nielsen. 2017. Absolute Quantification of Protein and mRNA Abundances Demonstrate Variability in Gene-Specific Translation Efficiency in Yeast. *Cell Syst.* 4:495–504.e5.

Lawless, C., S.W. Holman, P. Brownridge, K. Lanthaler, V.M. Harman, R. Watkins, D.E. Hammond, R.L. Miller, P.F. Sims, C.M. Grant, et al. 2016. Direct and Absolute Quantification of over 1800 Yeast Proteins via Selected Reaction Monitoring. *Mol. Cell. Proteomics.* 15:1309–1322. https://doi.org/10.1074/mcp.M115.054288
